# Pyomyositis After a Group C Streptococcal Toxic Shock Syndrome

**DOI:** 10.7759/cureus.54917

**Published:** 2024-02-26

**Authors:** Maria Pacheco, João C Rodrigues, Hugo R Almeida, Adriano Cardoso, Ana Teresa Moreira

**Affiliations:** 1 Internal Medicine, Hospital Sousa Martins, Unidade Local de Saúde Guarda, Guarda, PRT; 2 Internal Medicine, Hospital Universitario de Salamanca, Salamanca, ESP

**Keywords:** intravenous immunoglobulins (ivig), toxic-shock syndrome, atypical back pain, severe neutropenia, septic shock (ss), group c streptococcus, multiple pyomyositis

## Abstract

Pyomyositis is a bacterial infection deep within the muscles, often leading to multiple intramuscular abscesses. While historically linked with tropical regions, its incidence in temperate zones has been increasing, primarily due to factors such as immunosuppression. Typically, it manifests as a subacute infection, although when caused by Group C *Streptococcus* and resulting in toxic shock syndrome, it can lead to poorer outcomes. Here, we report a rare case of extensive multifocal bilateral pyomyositis in an immunocompetent young woman, preceded by toxic shock syndrome.

## Introduction

Pyomyositis is an infection of the muscles, usually initiated by the spread of bacteria through the bloodstream, often resulting in abscess formation within a single muscle, primarily caused by *Staphylococcus aureus* [1]; however, Group A *Streptococcus* may also be involved, and in rare cases, *Streptococcus* from Groups B, C, and G may be implicated [2]. Additionally, cases with Gram-negative bacteria have been described. It can progress to multiple intramuscular abscesses in 10-20% of the cases [3].

While traditionally associated with tropical climates, its occurrence in temperate regions has become more frequent, but still less common than in tropical regions [3]. It predominantly affects individuals with underlying conditions compromising their immune systems, such as malignancy, cirrhosis, HIV/AIDS, or intravenous drug use [4]. Common initial symptoms include fever, localized pain, and swelling within a single muscle, although multifocal infections can occur. It is a rare yet potentially serious condition capable of leading to septic shock, which can result in death [3].

## Case presentation

A previously healthy 39-year-old woman, working in an assisted living facility for the elderly, presented to the emergency department with a history of fever, fatigue, anorexia, and severe neck pain. Three weeks prior, she had begun experiencing lower back pain radiating to her hips and had sought medical attention from her family physician, who initially prescribed oral nonsteroidal anti-inflammatory drugs without complete resolution of symptoms. Subsequently, she sought further evaluation at a health center, where intramuscular anti-inflammatory medications were administered. There was no history of recent trauma.

On admission, she presented a temperature of 39.2°C (102.56°F), a heart rate of 132 beats/min, and a blood pressure of 75/52 mmHg, requiring aggressive intravascular resuscitation with limited response. Initial blood tests revealed severe leukopenia (0.2 × 109/L) with 10% neutrophils, along with elevated levels of C-reactive protein (37.11 mg/dL; normal range: <0.5 mg/dL), aspartate aminotransferase (1,058 U/L; normal range: 5-34 U/L), alanine aminotransferase (988 U/L; normal range: <45 U/L), creatine kinase (23,401 U/L; normal range: 30-200 U/L), creatinine (1.78 mg/dL; normal range: 0.6-1.3 mg/dL), and serum urea (59 mg/dL; normal range: 18-55 mg/dL), indicative of renal injury.

Due to septic shock, she was admitted to the ICU. Progressive scaling care and increasing needs were necessary, and she ended up requiring noradrenaline, terlipressin, hydrocortisone, and continuous renal replacement therapy due to renal dysfunction associated with shock and severe rhabdomyolysis. The initial choice of antibiotic fell upon piperacillin/tazobactam during the first 48 hours. Upon detection of Gram-positive bacteremia, vancomycin was initiated. Subsequently, due to sustained deterioration and the need for increased vasopressor support concurrent with severe pancytopenia, antibiotic coverage was expanded to include Gram-negative multidrug-resistant organisms with meropenem. Later, Group C Streptococci was isolated from sequential blood cultures, suggesting streptococcal toxic shock syndrome. Targeted antibiotic therapy and immunoglobulins were administered accordingly. As the creatine kinase decreased and subsequent improvement in renal function was observed, the patient no longer required renal replacement therapy.

Upon admission to the ICU, no visible cutaneous lesions were observed. On the 12th day of hospitalization, right gluteal swelling was noted. A CT scan was performed, which revealed a collection/necrotic area measuring 42 × 27 mm in the right gluteal muscles. An ultrasound-guided drainage was performed, but no agent was isolated. Subsequently, during the rest of the ICU stay, circular lesions appeared on both forearms, thighs, legs, the left temporal region, and the left supraorbital area. Initially presenting as erythematous, these lesions later exhibited a violaceous hue, with some becoming bullous. These lesions further developed into erythematous, scaly, pruritic plaques, particularly edematous, in the right hand and forearm and both legs and feet. Eventually, hyperpigmented skin areas emerged in the abscessed regions.

Due to persistent febrile neutropenia, prophylactic antibiotics and antifungals were prescribed alongside targeted antibiotic therapy. The choice of antibacterial agents was a combination of vancomycin and meropenem, for a total of 21 days. With the resolution of the septic shock, improvements in hepatic enzymology and neutropenia were observed. Blood tests indicated normalization of her white blood cell count on the 19th day after admission.

After 21 days of ICU treatment, the patient was transferred to the Department of Internal Medicine for further management.

**Figure 1:**
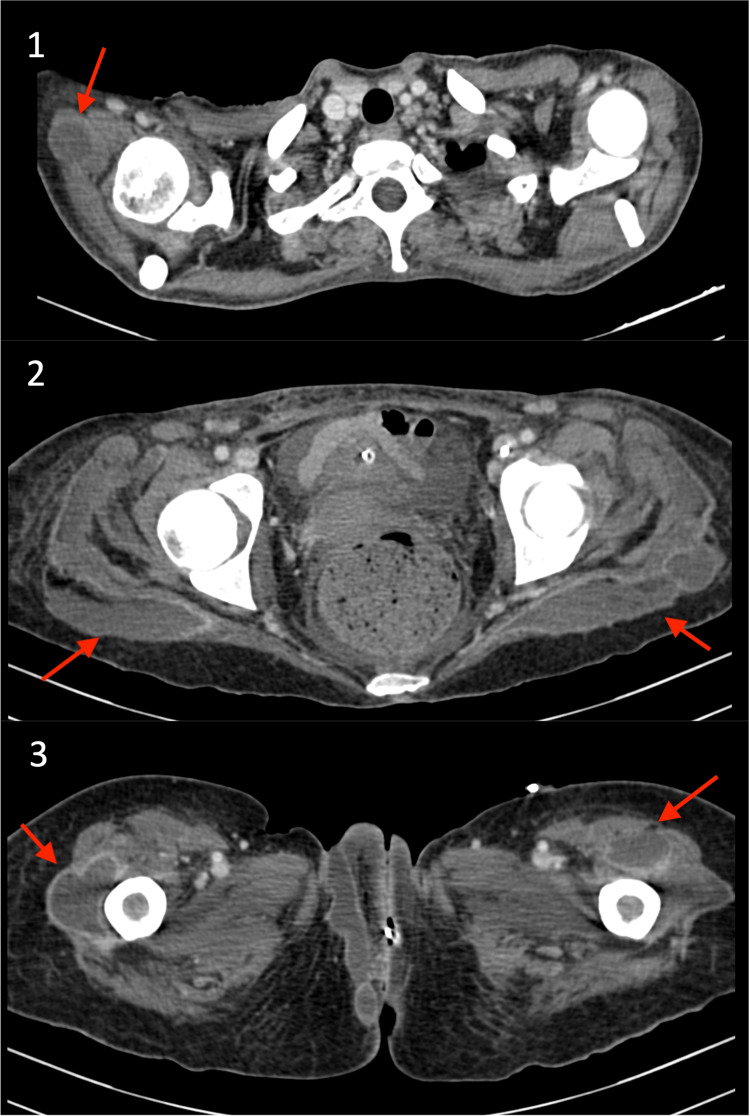
CT scan performed on the 30th day (1) Collection adjacent to the right humerus, measuring 26 × 24 mm. (2) In both gluteal regions, on the right measuring 86 × 28 mm and on the left measuring 106 × 32 mm. (3) On both femurs, on the right measuring 26 × 47 mm and on the left measuring 26 × 39 mm. The arrows show abscesses.

Following the first clinical observation in the ward, the previously described cutaneous findings evolved into painful erythematous plaques, some presenting also floating elastic consistency, especially in the lower limbs. A CT scan was performed, revealing a widespread increase in abscesses (Figure 1). Despite improvements in clinical parameters, recurrent fever and enlarging abscesses necessitated prolonged antibiotic treatment and multidisciplinary management.

**Figure 2:**
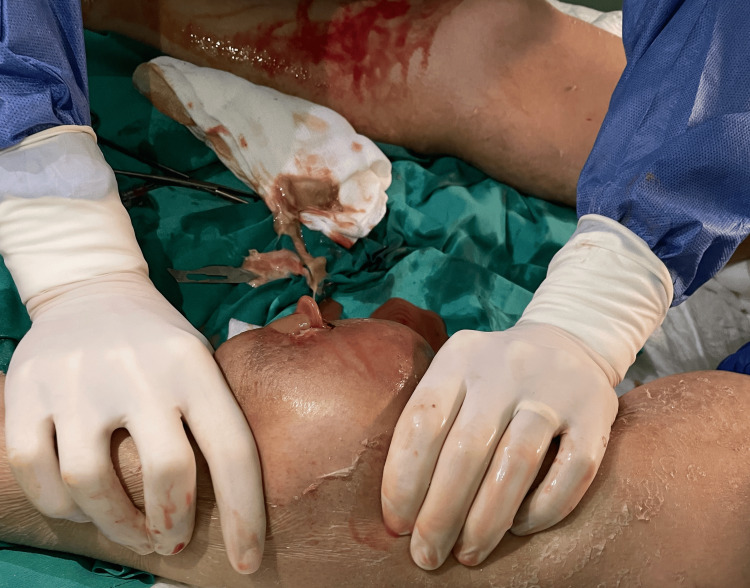
Bilateral surgical drainage of leg abscesses

Given the multiple subcutaneous and muscular abscesses, general surgery collaboration was necessary. This allowed for the drainage of the larger abscesses in both legs, leaving local drains due to the presence of scanty hematopurulent drainage (Figure 2). Pus sampling was conducted for microbiological analysis. However, no microbial agents were isolated.

**Figure 3:**
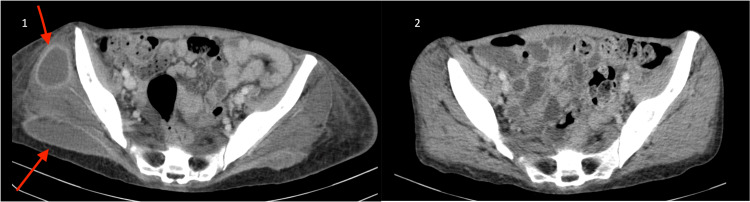
A CT scan was performed to evaluate the abscess in the gluteal region before and after drainage (1) A CT scan was performed before the drainage on the 40th day from the collection in the right gluteal region. (2) A CT scan was performed 60 days after discharge, showing no abscess in the gluteal muscle. The arrows show abscesses.

The patient demonstrated resolution of the abscesses, except for an intramuscular collection in the right thigh, which worsened. A repeat CT scan was conducted, confirming an enlargement of the abscess and prompting surgical drainage in the anterior region of the right thigh and calf areas (Figure 3), rather than surgical debridement.

Following multidisciplinary deliberation, the patient received a total of 50 days of broad-spectrum antibiotic therapy comprising vancomycin and meropenem, leading to significant improvement in the abscesses. No pathogenic agent was isolated.

During the hospitalization period, bone marrow studies, including myelograms, bone marrow biopsy, immunophenotyping of bone marrow blood, and culture of bone marrow blood; autoimmune studies; capillaroscopy; echocardiogram; skin biopsy; head and body CT scans; multiple ultrasound scans; several microbiological samples; and virus research, such as HIV, hepatitis, and other viral etiologies, were conducted. All other tests were negative for any underlying pathology except for the initial blood cultures.

The patient has shown improvement with treatment, and her blood tests indicated normalization of C-reactive protein on the 47th day of admission. Ultrasound and CT scan examinations revealed near-complete resolution of the collections. She was discharged to a rehabilitation center.

## Discussion

Streptococcal toxic shock syndrome, although typically associated with group A ß-hemolytic *Streptococcus*, can also involve groups C and D, as observed in this case [5]. The patient’s history of intramuscular injections preceding symptom onset suggests a potential point of entry for the infection.

The development of pyomyositis after septic shock due to *Streptococcus *species can occur due to several factors. *Streptococcus* species produce enzymes and toxins that facilitate their growth and penetration of muscle tissues while evading the host immune response [6,7]. Hematogenous spread, promoted by the release of proinflammatory cytokines, is another mechanism by which streptococcal bacteria disseminate through the bloodstream to reach distant sites, including skeletal muscles. Tissue damage due to toxins further exacerbates the inflammatory response [6,7], leading to the development of severe muscle infections like pyomyositis. However, pyomyositis may not manifest immediately after the onset of septic shock [8]. Initially, the focus may be on treating the primary infection and stabilizing the patient. As the patient’s condition improves, symptoms of pyomyositis, such as localized pain, swelling, and fever, become apparent [7,8].

In this case, the severe medullary stress resulted in significant severe neutropenia for at least 14 days. Hematogenous dissemination of toxins from *Streptococcus *[9], considering the extent of initial medullary involvement, likely caused a dysregulated leukocytic response to the infection, leading to the formation of multiple abscesses, resulting in this rare case of pyomyositis. Additionally, the prolonged stay in an ICU contributed to the critical illness myopathy associated with this underlying level of immunosuppression.

Pyomyositis requires a differential diagnosis, and it depends on the area involved and the agent that was isolated. That includes impetigo, cellulitis, and necrotizing fasciitis [10].

Imaging examinations aided in establishing the diagnosis. While MRI is considered the gold standard [11], CT proved to be the most useful examination for describing multiple lesions. Despite its limited sensitivity, ultrasound was valuable in monitoring the disease’s progression and follow-up.

Pyomyositis usually consists of three distinct stages. In the initial phase, there is progressive swelling and pain in the muscle due to bacterial infiltration, and there is no formation of an abscess. The condition progresses to the second stage, known as the suppurative phase, which typically occurs within 10-21 days and is characterized by the formation of an abscess and fever. At this point, the signs became clearer, and the majority of cases were diagnosed. The third and last stage is marked by the onset of septicemia, the development of abscesses (like a fluctuant muscle mass with erythema and edema) in multiple sites, and multi-organ dysfunction [12,13]. Our patient’s presentation was atypical for pyomyositis, highlighting the importance of recognizing atypical presentations for effective management.

## Conclusions

We present a rare case of bacterial pyomyositis in a young and previously healthy patient, without any identifiable immunosuppressive factors. This case underscores the rarity and complexity of bacterial pyomyositis, particularly in immunocompetent individuals. Overall, while staphylococcal infections can more frequently lead to pyomyositis, streptococcal infections are more commonly associated with this clinical progression, particularly in the context of septic shock, due to their pathogenicity and tissue affinity. Severe neutropenia, likely exacerbated by prolonged critical illness and rhabdomyolysis, contributed to the extensive abscess formation, highlighting the importance of recognizing atypical presentations of pyomyositis and initiating prompt, comprehensive management.
